# Polymorphic Aβ42 fibrils adopt similar secondary structure but differ in cross-strand side chain stacking interactions within the same β-sheet

**DOI:** 10.1038/s41598-020-62181-x

**Published:** 2020-03-31

**Authors:** Hongsu Wang, Lan Duo, Frederick Hsu, Christine Xue, Yoon Kyung Lee, Zhefeng Guo

**Affiliations:** Department of Neurology, Brain Research Institute, Molecular Biology Institute, University of California, Los Angeles, CA 90095 USA

**Keywords:** Protein aggregation, Alzheimer's disease

## Abstract

Formation of polymorphic amyloid fibrils is a common feature in neurodegenerative diseases involving protein aggregation. In Alzheimer’s disease, different fibril structures may be associated with different clinical sub-types. Structural basis of fibril polymorphism is thus important for understanding the role of amyloid fibrils in the pathogenesis and progression of these diseases. Here we studied two types of Aβ42 fibrils prepared under quiescent and agitated conditions. Quiescent Aβ42 fibrils adopt a long and twisted morphology, while agitated fibrils are short and straight, forming large bundles via lateral association. EPR studies of these two types of Aβ42 fibrils show that the secondary structure is similar in both fibril polymorphs. At the same time, agitated Aβ42 fibrils show stronger interactions between spin labels across the full range of the Aβ42 sequence, suggesting a more tightly packed structure. Our data suggest that cross-strand side chain packing interactions within the same β-sheet may play a critical role in the formation of polymorphic fibrils.

## Introduction

Formation of amyloid fibrils underlies a wide range of human disorders, including Alzheimer’s disease, Parkinson’s disease, and type 2 diabetes^1,2^. The amyloid fibrils formed by different proteins share some common properties such as binding of thioflavin T and Congo red, filamentous morphology, and, in most cases, a parallel β-sheet structure^3^. It has also been recognized that aggregation of the same protein may result in the formation of different amyloid fibrils, even in the same sample^4,5^. This phenomenon is called amyloid fibril polymorphism. In prion diseases, different fibril polymorphs may be the basis of different prion strains^6^. In Alzheimer’s disease, different fibril polymorphs are found in patients with different clinical subtypes^7,8^, and these polymorphs can propagate their own conformations in transgenic animal models, similarly as prion strains^9,10^.

We have a limited understanding about the structural diversity and detailed structural differences among different fibril polymorphs. Recent structural studies of Aβ42 amyloid fibrils shed some light on the structural details of Aβ42 fibril polymorphism. Using solid-state NMR, Xiao *et al*.^11^, Wälti *et al*.^12^, and Colvin *et al*.^13^ reported a common S-shaped structure for residues 17–42. It appears that the same fibril polymorph was obtained in these three studies. The packing interactions between protofilaments are also similar^12,13^. Using cryoEM and solid-state NMR, Gremer *et al*.^14^ determined the structure of Aβ42 fibrils that were prepared under acidic conditions, in contrast to the neutral pH used in Xiao *et al*.^11^, Wälti *et al*.^12^, and Colvin *et al*.^13^. The structure of Gremer *et al*.^14^ also shows an S-shaped structure for residues 17–42, but with different inter-protofilament packing. Schmidt *et al*.^15^ reported a cryoEM study of Aβ42 fibrils prepared under neutral pH, and the structure is different from the S-shaped fold. More recently, a structure of Aβ40 fibrils purified from Alzheimer’s brain tissue was determined using cryoEM, and the structure represents a polymorph that is different from fibrils prepared *in vitro*^16^. Although the protein was Aβ40, not Aβ42, the discovery that Aβ fibrils in the brain may adopt a brand-new structural polymorph^16^ further shows the importance of understanding fibril polymorphism.

**Figure 1:**
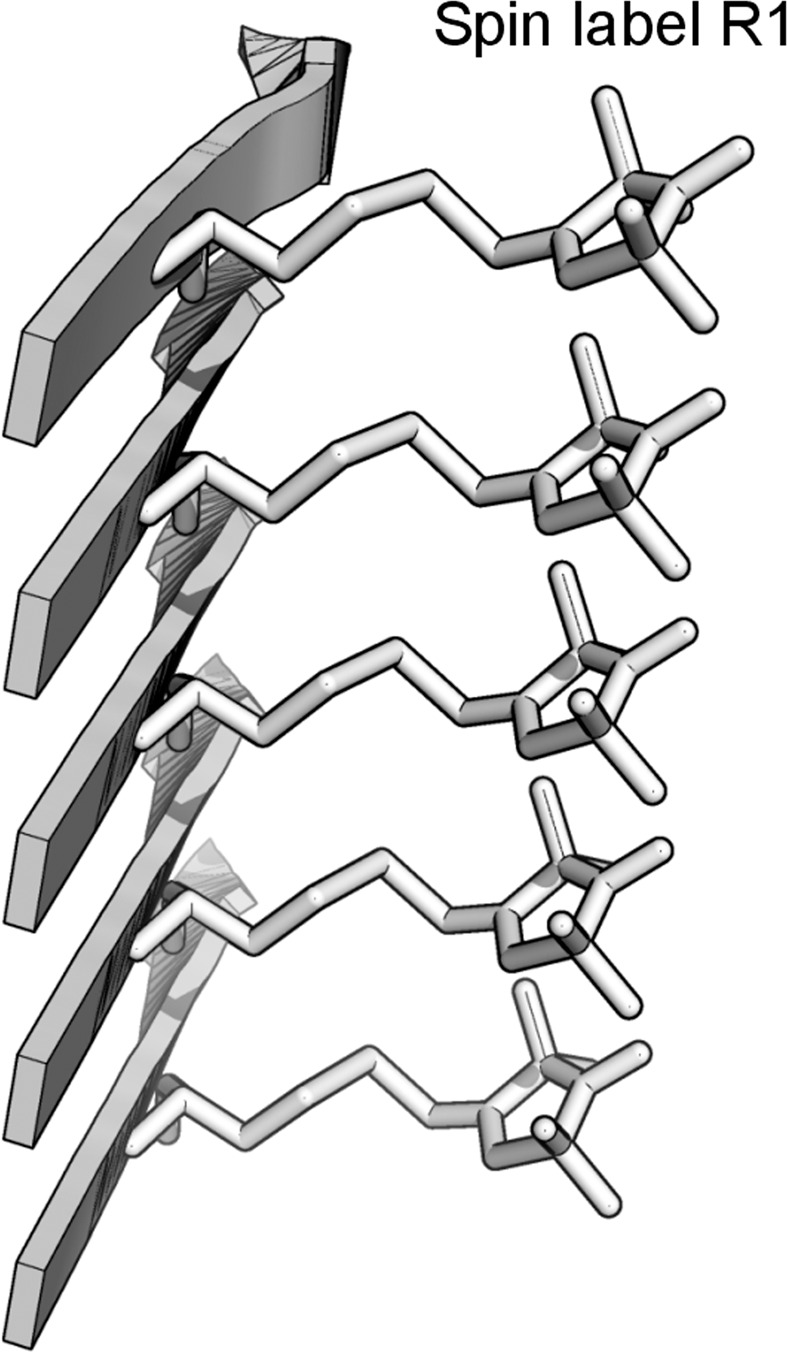
A schematic model of spin label stacking in a parallel in-register β-sheet. The stacking of spin label side chains leads to strong spin exchange interactions and the single-line EPR lineshape.

In this work, we aim to study the structural details in polymorphic Aβ42 fibrils using site-directed spin labeling and EPR spectroscopy. The strategy of site-directed spin labeling is to first mutate a residue of interest to cysteine, and then modify the cysteine side chain with a spin labeling reagent. The spin label side chain used in this work is named R1. EPR has been shown to be a powerful tool to reveal the parallel in-register β-sheet structure in the amyloid fibrils of a number of proteins including Aβ^17,18^, α-synuclein^19^, tau^20^, human prion^21^, and yeast prion Ure2^22^. In the parallel in-register β-sheet structure, the fibril samples of a protein spin-labeled at the same residue position leads to the cross-strand stacking of the spin label side chain. A model of this cross-strand stacking within the same β-sheet is shown in Fig. 1. The stacking of the spin labels leads to strong spin exchange interaction, a type of spin-spin interaction that occurs when orbitals of two spin centers overlap, due to the close distance between spin labels in the same β-sheet^23^. Furthermore, we have shown that quantitative analysis of the spin exchange interaction in combination with spin label scanning can reveal locations of β-strands and turns^24^. At secondary structure level, the EPR data on Aβ42 fibrils are consistent with results from solid-state NMR^11–13^ and cryoEM studies^14^. EPR data further revealed that side chains are more tightly packed in agitated Aβ40 fibrils than in quiescent Aβ40 fibrils. The strength of side chain packing interactions may determine the degree of β-sheet twist^25,26^, a distinguishing feature of some Aβ fibril polymorphs.

To study Aβ42 fibril polymorphism, we prepared Aβ42 fibrils with 42 singly spin-labeled Aβ42 variants, covering all 42 residue positions of the Aβ42 sequence. The Aβ42 fibrils were prepared in PBS at 37 °C either with or without agitation. We found that these two types of Aβ42 fibrils have distinct morphology. EPR data show that these fibril polymorphs are similar at secondary structure level, but differ in the strength of side chain packing. The difference in side chain packing may determine the degree of twist in β-sheets, forming the molecular basis of fibril polymorphism.

## Results and Discussion

### Agitation leads to formation of polymorphic Aβ42 fibrils

**Figure 2:**
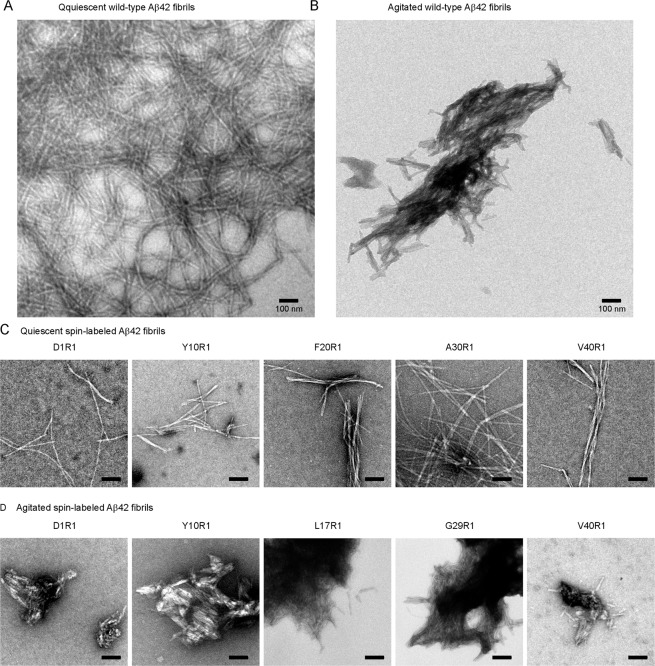
Transmission electron microscopy images of quiescent and agitated Aβ42 fibrils. Wild-type and spin-labeled Aβ42 fibrils were prepared in PBS buffer at 37 °C either without agitation (quiescent condition) or with shaking at 600 rpm. R1 represents the spin label. All scale bars are 100 nm.

We used two conditions to prepare Aβ42 fibrils in PBS buffer (pH 7.4) at 37 °C: an agitated condition with orbital shaking at 600 rpm, and a quiescent condition without any agitation. Agitation has previously been used to make polymorphic Aβ40 fibrils^17,27,28^. Transmission electron microscopy (TEM) studies show that Aβ42 formed long twisted fibrils under the quiescent condition, but short straight fibrils under agitated condition (Fig. 2, panels A and B). The agitated fibrils form bundles of laterally associated filaments. The quiescent fibrils are similar in morphology to other reported Aβ42 fibrils prepared in a neutral pH buffer without agitation^15^ or with slow rotation^11^. The morphology of the agitated Aβ42 fibrils is similar to that of agitated Aβ40 fibrils, as previously reported by our group^17^ and by the Wetzel group^27^.

To study the structural difference between quiescent and agitated fibrils, we used 42 spin-labeled Aβ42 variants for fibril preparation. Each variant is spin-labeled at a different residue position via site-directed spin labeling. Previously, in a kinetics study^29^, we have shown that all 42 spin-labeled mutants form fibrils. To check if spin-labeling affects the morphology of Aβ42 fibrils, we performed TEM studies on 5 spin-labeled Aβ42 mutants under the quiescent condition (Fig. 2C), and 5 mutants under the agitated condition (Fig. 2D). Generally speaking, the quiescent fibrils of spin-labeled Aβ42 are long and twisty, while the agitated fibrils are short and tend to form large bundles. These characteristics are similar to their wild-type counterparts. We also noticed some subtle differences among different spin-labeled Aβ42 mutants under the same conditions. At the TEM resolution we obtained, we could not determine how significant these subtle differences were. However, these differences do not obscure the fact that spin labeling does not change the main morphological feature of quiescent and agitated fibrils.

### EPR studies of spin-labeled Aβ42 fibrils reveal structural differences between quiescent and agitated fibrils

**Figure 3:**
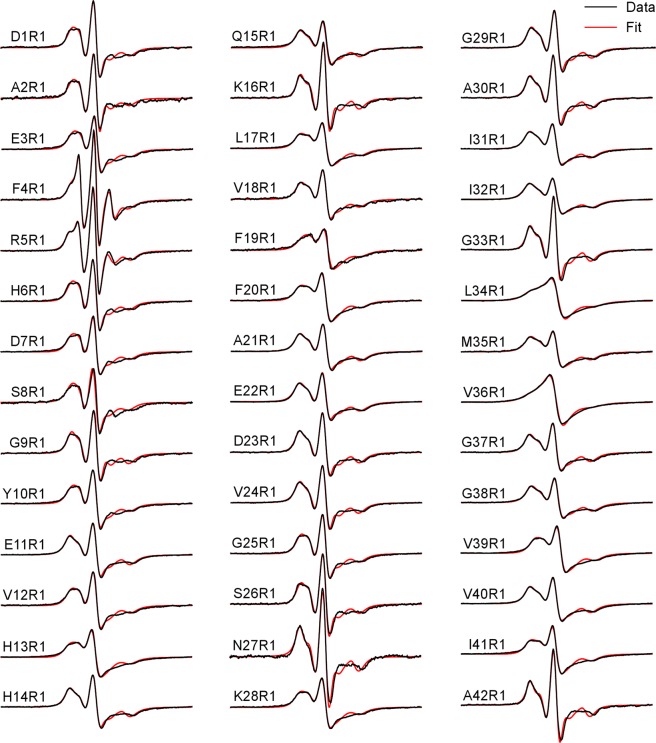
EPR spectra of quiescent fibrils of spin-labeled Aβ42 proteins. Experimental spectra are shown in black, and the best fits using spectral simulations are shown in red. R1 represents the spin label. Scan width is 200 G.

**Figure 4:**
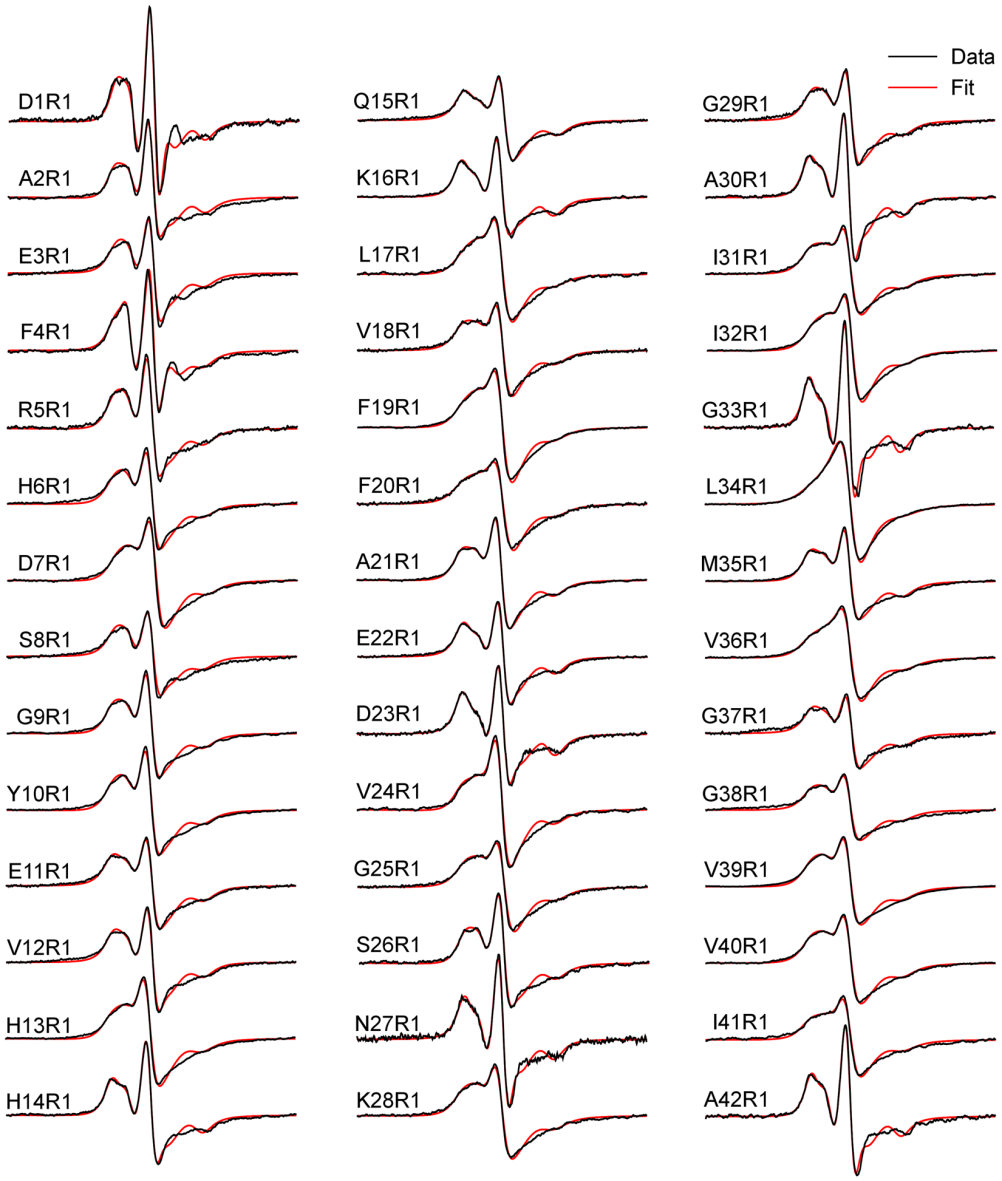
EPR spectra of agitated fibrils of spin-labeled Aβ42 proteins. Experimental spectra are shown in black, and the best fits using spectral simulations are shown in red. R1 represents the spin label. Scan width is 200 G.

For structural studies, we collected EPR spectra for each of the 42 spin-labeled Aβ42 fibril samples prepared under quiescent and agitated conditions. These EPR spectra are shown in Fig. 3 (quiescent fibrils) and Fig. 4 (agitated fibrils). The characteristic feature for both the quiescent and agitated Aβ42 fibrils is the single-line EPR spectrum, exemplified by the spectra of L34R1 and V36R1 of both quiescent and agitated conditions. The single-line feature is due to strong spin exchange interactions between spin label side chains, which stack on top of each other to form a spin label ladder in a parallel in-register β-sheet (Fig. 1)^23^. The strength of the spin exchange interaction is reflected in the EPR spectral lineshape. With weaker spin exchange interactions, the EPR spectra are characterized by bumps on the left half of the EPR spectra, such as the spectra of Y10R1 and E22R1. In the absence of spin exchange interactions or with very weak interactions, the EPR spectra show a typical three-line feature, as shown in the spectra of the two terminal residues: D1R1 and A42R1.

**Figure 5:**
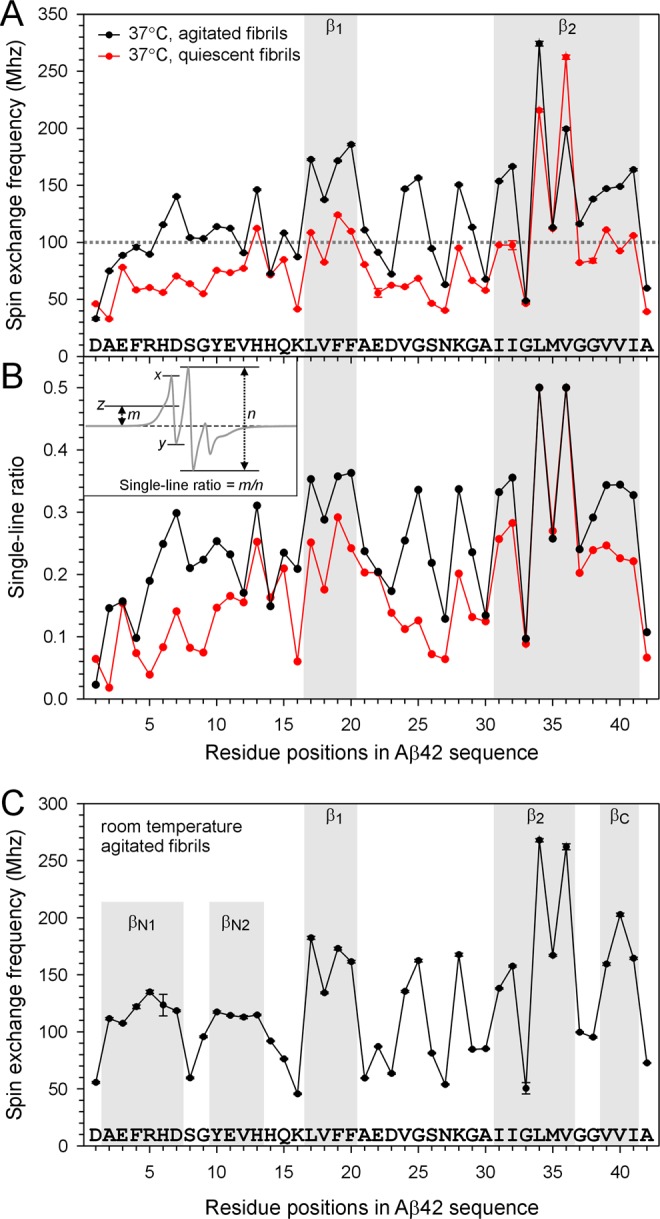
Quantitative analysis of spin exchange interactions in spin-labeled Aβ42 fibrils. (**A**) Spin exchange frequencies obtained from spectral simulations of quiescent and agitated fibrils are plotted as a function of residue positions. Note the similarity between quiescent and agitated fibrils, suggesting similar secondary structures. The agitated fibrils show overall stronger interactions, suggesting stronger packing. (**B**) Residue-specific single-line ratios for both quiescent and agitated fibrils. Inset shows how the single-line ratio is calculated. In the inset, line *z* is half-way between *x* and *y*. Distance *m* measures the upward shift for the center of the low-field peak relative to the baseline. For a spectrum without exchange interactions, *m* = 0. Distance *n* measures the amplitude of the center line. If the bumpy feature at the low-field line is completely smoothed out, distance *m* can not be determined and the single-line ratio is arbitrarily set at 0.5 (e.g., L34R1 and V36R1 in both fibril types). (**C**) Spin exchange frequencies of agitated Aβ42 fibrils prepared at room temperature, which have been published previously^18^ and are reproduced here for comparison with 37 °C agitated fibrils.

The structural basis for the strong spin exchange interaction is the parallel in-register β-sheet structure^23^. Therefore, quantitative analysis of spin exchange interaction can be used to identify locations with strong exchange interactions and assign them to β-strand structures^18,24,30^. The strength of spin exchange interaction is expressed as spin exchange frequency, which can be obtained by performing spectral simulations^31^. For spectral simulations, only three parameters were allowed to vary: spin exchange frequency, rotational correlation time and order parameter. The latter two parameters describe an anisotropic motional model for the spin label. The final values of the fitted parameters were obtained at the end of successful fitting without user intervention. The exchange frequency is plotted in Fig. 5A, and other fitted parameters are shown in Supplementary Table S1. It is likely that combinations of different values of these parameters may have led to similar fittings. To help evaluate this, the fitting program also calculates correlations between fitting parameters. The correlations between the exchange frequency and the other two fitting parameters are shown in Supplementary Fig. S1. In general, the exchange frequency has low correlations with either correlation time or order parameter when the spin exchange interaction is strong. This gives us high confidence for the EPR spectra with strong exchange interaction. When the exchange interaction is weak (<100 MHz), the correlation is higher with either correlation time or order parameter, suggesting that the exchange frequency values of these weaker exchange frequencies are less reliable. Only a handful of labeling positions gave correlation coefficients that are considered as large (>0.9) by the original developers of this spectral simulation program^31^. To further ensure that we have high confidence in the quantitative analysis, we used a previously developed empirical measurement for the spin exchange interaction, called single-line ratio^24^. The single-line ratio is calculated directly using the EPR spectral lineshape and is not subject to fitting errors. The values of the single-line ratio for spin-labeled Aβ42 are plotted in Fig. 5B. The general agreement between spin exchange frequency obtained from spectral simulations and the single-line ratio provides a cross validation for our quantitative data analysis.

The following points can be made from the results in Fig. 5A,B. First, the overall pattern of residue-dependent spin exchange frequency is very similar between quiescent and agitated fibrils. Because strong spin exchange interaction suggests highly ordered β-strand structure, we assigned consecutive residues of 3 or more with strong spin exchange interactions as β-strand structures. A spin exchange frequency of 100 MHz or higher is generally considered to have strong exchange interactions. We also considered relative strength in spin exchange interaction because some fibril types may show weaker exchange interactions across the whole sequence. Therefore, the pattern of residue-dependent spin exchange frequency is more important than the absolute values of exchange frequency when assigning β-strands. In agitated fibrils, two β-strand regions, β_1_ (17–20) and β_2_ (31–41), show high spin exchange interactions. In quiescent fibrils, these two regions show relatively higher exchange frequencies than the rest of the residue positions, although they are generally weaker than agitated fibrils. This suggests that both quiescent and agitated Aβ42 fibrils adopt similar secondary structures. Second, agitated fibrils show overall stronger spin exchange interactions across the entire Aβ42 sequence than quiescent fibrils, suggesting that agitated fibrils have stronger cross-strand side chain stacking within the same β-sheet. Third, the region spanning residues 34–36 has the strongest spin exchange interactions in both agitated and quiescent fibrils, suggesting that this segment is the most ordered region in Aβ42 fibrils. The strong exchange interactions at residues 34–36 are especially notable in quiescent fibrils, which show generally weak exchange interactions at residues other than 34–36. Fourth, there are some regions that show highly ordered structures in agitated fibrils, but not in quiescent fibrils. These regions include residues 6–7 and 24–25. Residues 24–25 are located in a long loop spanning residues 21 to 30. The strong spin exchange interactions of V24R1 and G25R1 suggest that this loop is highly ordered in agitated fibrils, but not in quiescent fibrils. The stronger spin exchange interactions at H6R1 and D7R1 suggest a more ordered N-terminal region in agitated fibrils.

The current study has some limitations. Several factors may affect the interpretation of spin exchange interaction. Strong spin exchange interaction requires both β-sheet backbone structure and fully labeled Aβ protein. We performed mass spectrometry to ensure high labeling efficiency at the beginning of fibril preparation, but spin labels could fall off during experiments and lead to weaker spin exchange interactions. Because a majority of the labeling sites show strong exchange interactions, the probability that the weak spin exchange interactions are caused by loss of spin labels is low. Sample heterogeneity may further complicate the data analysis. Aβ proteins are known to form polymorphic fibrils. We performed TEM on a subset of spin-labeled Aβ42 fibrils (Fig. 2). The TEM data show that spin-labeled Aβ fibrils maintain the main features of agitated and quiescent fibrils, but we cannot confirm whether or not they are composed of a single polymorph at the resolution we obtained. The presence of oligomers may provide another layer of uncertainty in data analysis. Some oligomers may be collected together with fibrils and contribute to the EPR spectra. With these caveats, the assignment of secondary structures using spin exchange interactions is somewhat speculative.

Previously, we have studied the structure of Aβ42 fibrils prepared at room temperature with agitation^18^. The spin exchange frequency plot is reproduced here for comparison (Fig. 5C). There are some notable differences. The N-terminal region in the room temperature fibrils shows a clear pattern of two β-strands due to low exchange interactions at residues 8–9. Contrastingly, these same residues show strong interactions in 37 °C fibrils, suggesting an overall strong order for the N-terminal region without clear distinction of β-strands and turns. In the C-terminal region, residues 37 and 38 show weak spin exchange interactions in room temperature agitated fibrils, allowing us to assign them to a turn. In 37 °C agitated fibrils, both residues 37 and 38 show strong exchange interactions, supporting a long C-terminal β-strand. The loop region covering residues 21 to 30 is highly ordered in both 37 °C and room temperature fibrils, with residues 24, 25, and 28 showing strong spin exchange interactions on par with β-strand residues.

There appears to be some growing consensus that the structure of the protofilament in polymorphic fibrils adopts similar structures, and different packing of protofilament leads to fibril polymorphism. The protofilament of α-synuclein fibrils was found to adopt similar secondary structure in four recent reports that used solid-state NMR^32^ and cryoEM^33–35^. The paired helical and straight tau filaments from Alzheimer’s disease have different inter-protofilament packing, but similar protofilament structure^36^. Studies of Aβ42 fibrils also show similar S-shaped fold at the protofilament level, with different packing between protofilaments for fibrils prepared under different conditions^11–14^. Our EPR data show that quiescent and agitated Aβ42 fibrils adopt similar secondary structures, supporting a common protofilament structure in polymorphic fibrils (Fig. 5). At the same time, EPR data also reveal different strength of cross-strand side chain stacking interactions within the same β-sheet. This finding is credited to the sensitivity of EPR measurements to not only static structure, but also backbone and side chain dynamics. Side chain interactions in the β-sheet have been shown to be a determining factor of fibril twist^25,26^, a feature that is both distinguishing and most recognizable for different fibril polymorphs. While backbone hydrogen bonds always favor flat β-sheets, side chain interactions favor twisted β structures^37^. The polymorphic nature of fibril formation likely results from difference in side chain interactions that lead to the formation of polymorphic fibril nuclei, which grow to form mature polymorphic fibrils.

## Materials and Methods

### Preparation of Aβ42 proteins and spin labeling

The constructs of wild-type GroES-ubiquitin-Aβ42^38^ and the deubiquitylating enzyme Usp2-cc^39^ were kindly provided by Dr. Il-Seon Park at Chosun University (South Korea) and Dr. Rohan T. Baker at Australian National University (Australia). Mutagenesis to introduce single cysteine mutations has been described previously^18^. Expression and purification of the Aβ fusion protein was performed as previously described^30,40^. Full-length Aβ was obtained by cleaving off the fusion protein with Usp2-cc as described^41^. For spin labeling, the spin labeling reagent MTSSL (1-oxyl-2,2,5,5-tetramethylpyrroline-3-methyl methanethiosulfonate, AdipoGen Life Sciences) was used. Detailed protocols have been previously described^30,41^. Spin labeling efficiency was checked with mass spectrometry and only samples with >95% labeling efficiency were used in subsequent studies. All spin-labeled Aβ42 proteins were lyophilized and stored at −80 °C.

### Preparation of Aβ42 fibrils

Lyophilized Aβ42 powder was first dissolved in 100% 1,1,1,3,3,3 hexafluoro-2-propanol (HFIP) to 100 µM final Aβ concentration, and then incubated at room temperature for 24 h with shaking at 1000 rpm. HFIP was evaporated overnight in the fume hood. To prepare fibrils, HFIP-treated Aβ was dissolved in CG buffer (20 mM CAPS, 7 M guanidine hydrochloride, pH 11) to 1 mM concentration, then was diluted 20-fold to PBS buffer (50 mM phosphate, 140 mM NaCl, pH 7.4) so that the final Aβ concentration is 50 µM. Then Aβ samples were placed either in a thermomixer (Eppendorf Thermomixer R) with shaking speed of 600 rpm at 37 °C (for agitated condition) or in a 37 °C incubator without agitation (for quiescent condition). Fibril growth was monitored with thioflavin T fluorescence. After thioflavin T fluorescence reached plateau (5–10 days), fibrils were collected by centrifugation at 20,000 g for 20 min. The fibril pellet was surface-washed twice with PBS buffer.

### Transmission electron microscopy

For electron microscopy, 5 µL of Aβ fibril samples were applied on glow-discharged copper grids (400 mesh formvar/carbon film, Ted Pella) and stained with 2% uranyl acetate. The grids were examined using a FEI T12 electron microscope with an accelerating voltage of 120 kV.

### EPR spectroscopy and spectral simulations

For EPR measurements, the fibril pellet was resuspended in approximately 20 µL of PBS buffer and then loaded into glass capillaries (VitroCom) sealed at one end. EPR spectra were collected at X-band using a Bruker EMX spectrometer fitted with the ER4102ST cavity at room temperature. Modulation amplitude was optimized for individual spectrum (typically 4 G). Typically 10 to 30 scans were averaged for each EPR spectrum. To quantify the strength of spin exchange interactions, spectral simulations were performed using the program MultiComponent, written by Dr. Christian Altenbach at University of California Los Angeles. A microscopic order macroscopic disorder model was used to describe the motion of spin label^31^. A least-squares fit of the user-defined spectral parameters was performed using the Levenberg-Marquardt algorithm. Detailed fitting procedure has been previously described^30^. For all the fits, the magnetic tensor *A* and *g* were set as *A*_*xx*_ = 6.2, *A*_*yy*_ = 5.9, *A*_*zz*_ = 37.0, and *g*_*xx*_ = 2.0078, *g*_*yy*_ = 2.0058, *g*_*zz*_ = 2.0023 as described previously^42^. An anisotropic model of motion was used for R1 by including an order parameter (*S*). For anisotropic simulations, the diffusion tilt angles were fixed to (α,β,γ) = (0,36°,0) for z-axis anisotropy as previously described^42^. The number of fitted parameters was kept at a minimum. We found that satisfactory fits were obtained with three fitted parameters: rotational diffusion constant (*R*) and order parameter (*S*) to describe the motion of the spin label, and Heisenberg exchange frequency (ω) to represent the rate of spin exchange. Rotational correlation time (τ) was calculated using τ = 1/(6*R*). For residue 4 of agitated fibrils and residues 4 and 5 of quiescent fibrils, a second spectral component with isotropic motion and without spin exchange interactions was also used to represent a locally disordered state. The fitting procedure was allowed to converge without intervention to obtain the spin exchange frequency (Fig. 5). All fitted parameters are reported in Supplementary Table S1, and correlations between exchange frequency and other fitting parameters are shown in Supplementary Fig. S1.

## Supplementary information

Supplementary information.

## Data Availability

Data available from the corresponding author upon reasonable request.
