# Supplementary material for: Polymorphic Aβ42 fibrils adopt similar secondary structure but differ in cross-strand side chain stacking interactions within the same β-sheet
Source: Sci Rep. 2020 Mar 31;10:5720. doi: 10.1038/s41598-020-62181-x (PMC7109039; doi:10.1038/s41598-020-62181-x)
Supplement: Supplementary file 1 — Supplementary information. [file 41598_2020_62181_MOESM1_ESM.pdf]

Polymorphic Aβ42 fibrils adopt similar secondary structure but differ in cross-strand side chain stacking interactions within the same β-sheet

Hongsu Wang, Lan Duo, Frederick Hsu, Christine Xue, Yoon Kyung Lee, and Zhefeng Guo*

Department of Neurology, Brain Research Institute, Molecular Biology Institute, University of California, Los Angeles, CA 90095, USA.

*To whom correspondence should be addressed: Zhefeng Guo, Department of Neurology, University of California, Los Angeles, 710 Westwood Plaza, Los Angeles, CA 90095. Phone: (310) 439-9843; E-mail: zhefeng@ucla.edu

Supplementary Information

Table S1. Values of Heisenberg exchange frequency (ω), rotational correlation time (τ) and order parameter (*S*) from spectral simulations. For quiescent fibrils, residue 4 has 78.6% for the major component and 21.4% for the minor component; residue 5 has 72.9% for the major component and 27.1% for the minor component. For agitated fibrils, residue 4 has 83.4% for the major component and 16.6% for the minor component.

Labeling sites	Quiescent fibrils			Agitated fibrils		
	ω (MHz)	τ (ns)	*S*	ω (MHz)	τ (ns)	*S*
1	46.0 ± 0.7	4.38 ± 0.09	0.44 ± 0.02	33.0 ± 1.0	4.61 ± 0.13	0.39 ± 0.03
2	32.8 ± 0.8	4.05 ± 0.10	0.41 ± 0.02	74.9 ± 0.6	5.57 ± 0.08	0.46 ± 0.01
3	78 ± 0.5	4.29 ± 0.07	0.51 ± 0.01	88.5 ± 0.7	5.41 ± 0.08	0.33 ± 0.02
4 (major component)	58.2 ± 0.8	0.75 ± 0.03	0.92 ± 0.01	95.6 ± 1.6	3.04 ± 0.08	0.70 ± 0.01
4 (minor component)	-	1.20 ± 0.02	-	-	2.45 ± 0.05	-
5 (major component)	60.2 ± 0.6	2.88 ± 0.16	0.85 ± 0.01	89.4 ± 0.4	5.37 ± 0.05	0.41 ± 0.01
5 (minor component)	-	2.28 ± 0.02	-	-	-	-
6	55.9 ± 0.6	4.11 ± 0.09	0.50 ± 0.01	115.4 ± 0.5	5.77 ± 0.06	0.35 ± 0.01
7	70.4 ± 0.6	4.71 ± 0.08	0.46 ± 0.01	140.0 ± 0.6	7.17 ± 0.06	0.33 ± 0.01
8	63.6 ± 0.8	4.20 ± 0.11	0.45 ± 0.02	104.0 ± 0.7	4.25 ± 0.07	0.54 ± 0.01
9	54.9 ± 0.4	1.05 ± 0.05	0.86 ± 0.01	103.3 ± 0.4	5.82 ± 0.05	0.45 ± 0.01
10	75.4 ± 0.5	4.72 ± 0.07	0.46 ± 0.01	113.7 ± 0.5	6.12 ± 0.05	0.33 ± 0.01
11	73.4 ± 0.3	1.37 ± 0.03	0.90 ± 0.00	112.2 ± 0.4	5.08 ± 0.05	0.65 ± 0.00
12	77.2 ± 0.4	5.29 ± 0.06	0.47 ± 0.01	90.7 ± 0.5	5.05 ± 0.06	0.55 ± 0.01
13	112.2 ± 0.5	6.51 ± 0.06	0.47 ± 0.01	146.1 ± 0.7	7.89 ± 0.07	0.29 ± 0.01
14	71.3 ± 0.3	1.36 ± 0.03	0.90 ± 0.00	72.6 ± 0.3	4.66 ± 0.06	0.63 ± 0.01
15	84.8 ± 0.3	1.57 ± 0.03	0.87 ± 0.00	108.0 ± 0.4	5.09 ± 0.06	0.74 ± 0.00
16	41.5 ± 0.5	1.23 ± 0.06	0.88 ± 0.01	87.2 ± 0.3	2.30 ± 0.04	0.84 ± 0.00
17	108.4 ± 0.4	5.18 ± 0.05	0.67 ± 0.00	172.6 ± 0.8	7.66 ± 0.07	0.37 ± 0.01
18	82.5 ± 0.3	3.49 ± 0.06	0.69 ± 0.00	137.3 ± 0.6	6.88 ± 0.07	0.57 ± 0.00
19	124.0 ± 0.8	4.82 ± 0.07	0.51 ± 0.01	171.4 ± 0.7	7.75 ± 0.07	0.34 ± 0.01
20	109.6 ± 0.4	6.39 ± 0.05	0.56 ± 0.00	185.8 ± 1.0	8.15 ± 0.10	0.57 ± 0.00
21	80.4 ± 0.5	1.33 ± 0.01	0.92 ± 0.00	110.8 ± 0.3	7.16 ± 0.05	0.48 ± 0.01
22	55.7 ± 3.9	1.85 ± 0.11	0.95 ± 0.00	91.2 ± 0.3	4.70 ± 0.05	0.71 ± 0.00
23	62.3 ± 0.3	1.27 ± 0.04	0.89 ± 0.01	72.0 ± 0.3	1.69 ± 0.04	0.89 ± 0.00
24	61.0 ± 0.6	1.01 ± 0.02	0.92 ± 0.01	146.7 ± 0.7	10.64 ± 0.13	0.01 ± 0.16
25	68.3 ± 0.4	4.26 ± 0.07	0.56 ± 0.01	156.3 ± 0.7	6.70 ± 0.07	0.53 ± 0.00
26	46.5 ± 0.6	4.27 ± 0.11	0.54 ± 0.01	94.4 ± 0.4	5.92 ± 0.06	0.45 ± 0.01
27	40.2 ± 0.6	1.52 ± 0.09	0.86 ± 0.01	62.8 ± 0.4	3.70 ± 0.08	0.68 ± 0.01
28	95.0 ± 0.4	5.61 ± 0.05	0.53 ± 0.01	150.4 ± 0.6	6.56 ± 0.06	0.53 ± 0.00
29	66.4 ± 0.3	1.35 ± 0.04	0.88 ± 0.00	113.1 ± 0.5	5.07 ± 0.06	0.58 ± 0.00
30	57.8 ± 0.4	1.33 ± 0.04	0.91 ± 0.01	67.6 ± 0.3	5.53 ± 0.06	0.57 ± 0.01
31	97.7 ± 0.2	1.68 ± 0.02	0.88 ± 0.00	153.6 ± 0.6	6.47 ± 0.06	0.63 ± 0.00
32	97.5 ± 6.3	1.59 ± 0.10	0.94 ± 0.00	166.5 ± 0.6	7.39 ± 0.06	0.44 ± 0.01
33	46.2 ± 0.5	1.35 ± 0.04	0.91 ± 0.01	48.6 ± 0.5	1.32 ± 0.05	0.91 ± 0.01
34	215.6 ± 1.3	13.51 ± 0.28	0.69 ± 0.00	274.1 ± 2.1	16.02 ± 0.42	0.55 ± 0.01
35	112.1 ± 0.3	2.74 ± 0.04	0.81 ± 0.00	113.0 ± 0.4	5.15 ± 0.05	0.67 ± 0.00
36	262.3 ± 1.8	18.90 ± 0.49	0.61 ± 0.00	199.4 ± 1.0	10.49 ± 0.15	0.58 ± 0.00
37	82.1 ± 0.3	1.80 ± 0.04	0.88 ± 0.00	116.2 ± 0.7	4.45 ± 0.08	0.67 ± 0.00
38	83.9 ± 2.0	1.50 ± 0.06	0.94 ± 0.00	137.8 ± 0.8	5.02 ± 0.07	0.60 ± 0.01
39	110.9 ± 0.4	5.43 ± 0.05	0.59 ± 0.00	147.1 ± 0.6	5.79 ± 0.05	0.56 ± 0.00
40	92.3 ± 0.2	2.11 ± 0.03	0.85 ± 0.00	148.8 ± 0.5	5.98 ± 0.05	0.59 ± 0.00
41	105.8 ± 0.4	6.83 ± 0.06	0.54 ± 0.01	163.7 ± 0.9	8.15 ± 0.10	0.57 ± 0.00
42	39.1 ± 0.5	1.49 ± 0.08	0.88 ± 0.01	59.7 ± 0.4	3.78 ± 0.08	0.68 ± 0.01

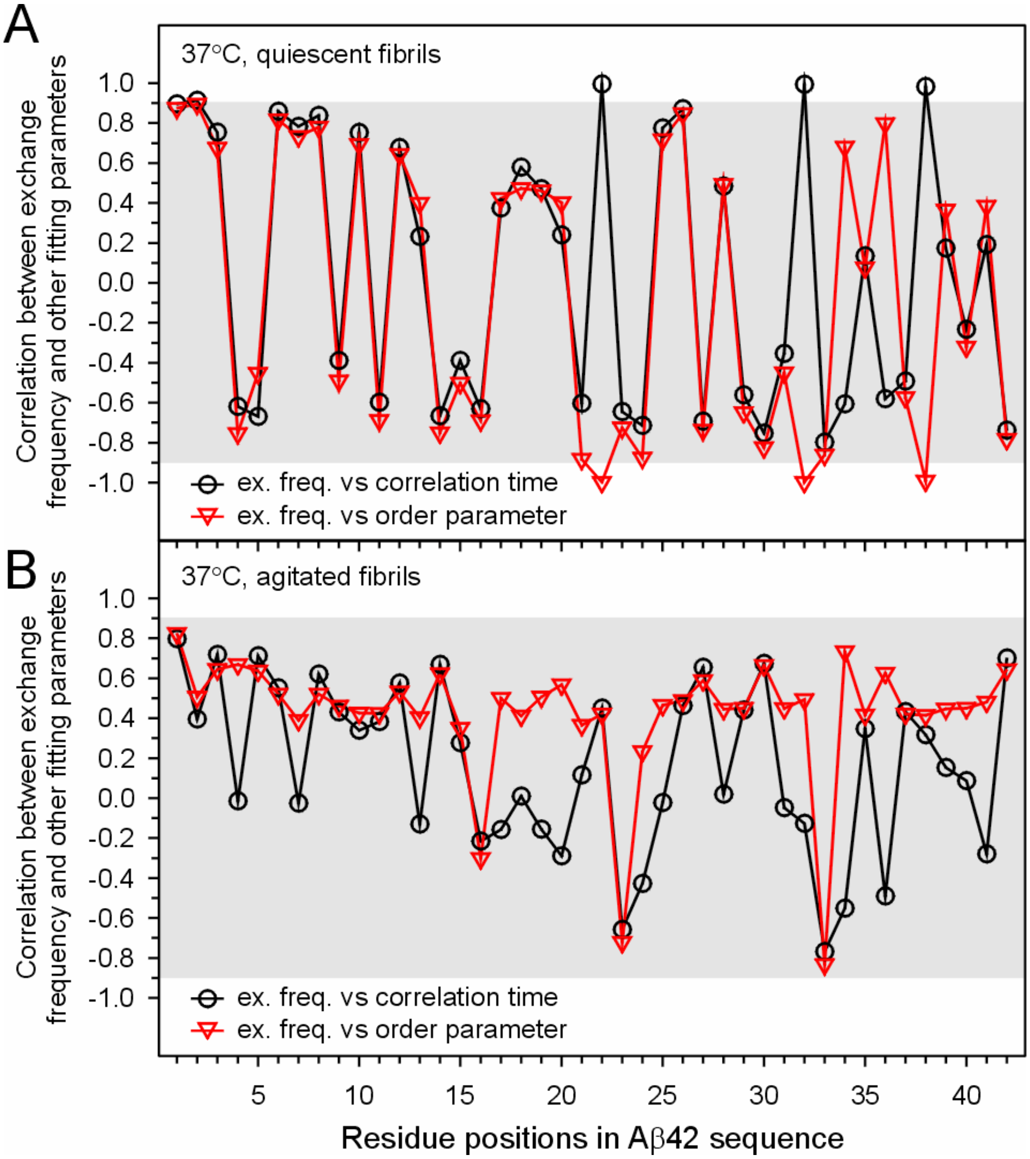

Figure S1. Correlations between Heisenberg exchange frequency (ω) and the two other fitted parameters (correlation time (τ) and order parameter (*S*)) for quiescent (*A*) and agitated (*B*) fibrils. Shaded boxes highlight the data points with correlations of <0.9 or >-0.9.
